# Pharmacokinetic and pharmacodynamic profiles of a novel phospholipid-aspirin complex liquid formulation and low dose enteric-coated aspirin: results from a prospective, randomized, crossover study

**DOI:** 10.1007/s11239-022-02687-5

**Published:** 2022-08-29

**Authors:** Francesco Franchi, David J. Schneider, Jayne Prats, Weihong Fan, Fabiana Rollini, Latonya Been, Heidi S. Taatjes-Sommer, Deepak L. Bhatt, Efthymios N. Deliargyris, Dominick J. Angiolillo

**Affiliations:** 1 Division of Cardiology, University of Florida College of Medicine – Jacksonville, 655 West 8th Street, Jacksonville, FL 32209 USA; 2 Department of Medicine, Cardiovascular Research Institute, The University of Vermont, Burlington, VT USA; 3 Elysis LLC, Carlisle, MA USA; 4 PLx Pharma, Inc., Sparta, NJ USA; 5 Brigham and Women’s Hospital, Harvard Medical School, Boston, MA USA; 6 Science and Strategy Consulting Group, Basking Ridge, NJ USA

**Keywords:** Aspirin, Pharmacodynamic, Pharmacokinetic, Platelet, PL-ASA

## Abstract

Low dose enteric-coated aspirin (EC-ASA) is routinely used for secondary cardiovascular event prevention. However, absorption of EC tablets is poor, which can result in subtherapeutic antiplatelet effects. Phospholipid-aspirin liquid filled capsules (PL-ASA) are a novel FDA-approved immediate-release formulation designed to reduce gastrointestinal (GI) injury by limiting direct contact with the stomach lining. We compared the pharmacokinetic (PK) and pharmacodynamic (PD) profiles of PL-ASA versus EC-ASA at a low dose. This randomized, open-label, crossover study assessed PK and PD following a single 81-mg dose of PL-ASA versus EC-ASA under fasting conditions in 36 volunteers without cardiovascular disease between 18 and 75 years of age. Volunteers were randomly assigned 1:1 to either PL-ASA then EC-ASA or vice versa with a minimum 14-day washout. Assessments included PK parameters for acetylsalicylic acid and salicylic acid, platelet aggregation in response to arachidonic acid (AA), and serum thromboxane B2 (TxB_2_) assessments over 24 h. PL-ASA was rapidly absorbed. PL-ASA reached T_max_ 3 h earlier (1.01 vs. 4.00 h, p < 0.0001), with almost double the C_max_ (720 vs. 368 ng/mL, p < 0.0001) and overall 44% higher exposure of acetylsalicylic acid (AUC_0-t_: 601 vs. 416 h*ng/mL, p = 0.0013) compared with EC-ASA. Within 1 h of dosing, PL-ASA achieved significantly lower residual platelet aggregation, which persisted for the full 24 h (median AA-LTA was 47% with PL-ASA vs. 80.5% with EC-ASA; p = 0.0022 at hour-24). Treatment with PL-ASA also resulted in significantly lower serum TxB_2_ concentrations at each time point compared with EC-ASA (all p-values < 0.05). PL-ASA resulted in faster and more complete aspirin absorption paralleled by more prompt and potent platelet inhibition compared with EC-ASA after a single 81 mg dose. PL-ASA represents an attractive novel aspirin formulation for the secondary prevention of cardiovascular events.

*Clinical Trial Registration* ClinicalTrials.gov identifier: NCT04811625.

**Supplementary Information:**

The online version contains supplementary material available at 10.1007/s11239-022-02687-5.

## Highlights

- Lifelong low dose aspirin is the foundational antithrombotic therapy for secondary prevention in patients with atherosclerotic cardiovascular disease.
- Plain aspirin is associated with gastric injury, and enteric-coated aspirin formulations are commonly used based on the proposed reduced risk for gastrointestinal injury. However, due to unpredictable absorption and bioavailability, enteric formulations may result in suboptimal antiplatelet effects.
- A novel liquid capsule phospholipid aspirin (PL-ASA) formulation specifically designed to limit direct contact with the gastric mucosa has been determined to be bioequivalent to immediate-release aspirin, with better absorption and more predictable and complete antiplatelet effect compared to EC-ASA at the 325 mg dose.
- The PL-ASA 81 mg PK-PD profile is consistent with previous PK-PD studies with 325 mg and demonstrates rapid, predictable, and potent absorption and antiplatelet effects.
- The 81 mg PL-ASA formulation with optimized PK-PD characteristics represents an attractive alternative to EC-ASA for secondary prevention of cardiovascular events.

## Introduction

Atherosclerotic cardiovascular disease (ASCVD) is the leading cause of morbidity and mortality globally, and lifelong medical therapy including antithrombotic protection is recommended. However, recurrent events in patients with ASCVD still occur, including an estimated 335,000 myocardial infarctions (MI), and 185,000 strokes on annual basis in the U.S. alone [1]. Aspirin remains a cornerstone therapy for secondary ASCVD prevention [2–7], although its role in primary prevention is now limited to certain high-risk patients [8, 9].

Although effective as an antiplatelet agent and recommended as a first-line therapy for patients with ASCVD, plain, immediate-release aspirin is associated with gastrointestinal (GI) injury, even at low doses [10]. One component of gastric injury is attributable to a direct, local effect on the gastric mucosa. Although the relative contributions of systemic and local injury have not been fully determined, local injury is thought to play an important role. Plain aspirin associates with the protective hydrophobic phospholipid layer of the stomach mucosa and changes the nature of the layer, increasing the ability of stomach acid to erode and attack the lining and allow local acute injury [11]. In an effort to reduce GI injury, a delayed release formulation enteric-coated aspirin (EC-ASA) was developed and is currently widely used for secondary cardiovascular event prevention, usually at doses of 75–100 mg daily. However, whether EC-ASA reduces GI toxicity remains debated [12, 13]. Further, EC-ASA is associated with considerable variability in aspirin absorption and resultant antiplatelet effect [14–16].

PL-ASA is a novel phospholipid-aspirin complex administered in liquid filled capsules designed to mitigate gastric injury by limiting direct contact of aspirin with the stomach lining [17]. The pre-associated phospholipid-aspirin complex (PLxGuard™ delivery platform, PLx Pharma, Sparta, NJ) remains together in low pH environments, such as the stomach, thereby limiting free aspirin contact with the gastric mucosa [18]. A clinical endoscopy study in healthy subjects randomized to 7 days of either PL-ASA or plain aspirin at a 325 mg dosing regimen showed a 47.4% reduction in the composite of gastroduodenal erosions or ulcers, and 71% fewer ulcers with the novel formulation [10].

Previous studies have also established the bioequivalence of 325 mg PL-ASA to immediate-release aspirin according to FDA bioequivalence criteria [19]. An additional study comparing the 325 mg dose in obese subjects with diabetes mellitus validated both the pharmacokinetic (PK) and pharmacodynamic (PD) bioequivalence of PL-ASA and plain, immediate-release aspirin (IR-ASA) and also demonstrated the delayed and reduced aspirin absorption associated with 3-day dosing of EC-ASA that resulted in reduced antiplatelet effects compared with either plain or PL-ASA formulations [20]. PL-ASA is approved as an immediate-release, over-the-counter aspirin formulation by the FDA.

The current study assessed the PK (absorption and distribution) and PD (antiplatelet) profiles of an 81 mg dose of the approved PL-ASA formulation, with comparison to EC-ASA.

## Methods

### Study design and study population

This first in human study was a randomized, open-label, crossover study assessing PK and PD profiles following treatment with a single 81 mg dose of PL-ASA or EC-ASA under fasting conditions in 36 subjects without known ASCVD or other acute medical conditions. The study was conducted in accordance with the principles of the Declaration of Helsinki in place at the time of study conduct, and in compliance with the International Council for Harmonisation (ICH) E6 Guideline for Good Clinical Practice (GCP) (European Medicines Agency [EMA]/Committee for Medicinal Products for Human Use [CHMP]/ICH/135/1995). The study initially started with an age limit of 50 to 75 years of age, but after enrollment of the first 26 subjects, the protocol was amended to allow inclusion of all adults from 18 to 75 years of age to facilitate enrollment during the coronavirus pandemic. The study was approved by WCG™ Institutional Review Board and conducted at the University of Florida, Jacksonville between April 29, 2021 (first screening) and September 15, 2021 (last follow-up). Study subjects were randomly assigned at a 1:1 ratio to either PL-ASA followed by EC-ASA or EC-ASA followed by PL-ASA with a minimum 14-day washout period between the two study drug administrations. Subjects refrained from eating or drinking (except for water) for at least 10 h overnight and abstained from alcohol for 48 h prior to dosing. A standard lunch and dinner were provided 4 h and 10 h after study drug administration (within 30 min after the 4 and 10 hour blood draws) respectively, and a snack was given after the hour 12 blood draw. Then subjects refrained from eating and drinking (except for water) until they completed their hour 24 blood draw on Day 2, after which they were allowed to eat and drink fluids but continued abstinence from alcohol for another 24 h (total 48 h after the drug administration). Both study drugs were swallowed whole and not chewed under direct observation by the study coordinator, with 240 mL water provided at the time. After each drug administration, an oral cavity inspection was conducted to make sure the drug was taken. Inclusion and exclusion criteria are shown in Supplemental Table 1.

A critical eligibility criterion for enrollment was a screening blood test result of ≥ 60% arachidonic acid (AA)-induced platelet aggregation using light transmission aggregometry (LTA), indicating normal platelet function. If a subject’s assay for screening indicated a level of aggregation below 60%, the subject could be re-screened at the discretion of the investigator, after a few additional days of abstaining from aspirin or other nonsteroidal anti-inflammatory drugs (NSAID). Importantly, this eligibility criterion was also required to be met prior to the dosing of the first and second phases of the study.

### Pharmacokinetic assessments

Blood samples for PK assessments were collected at baseline (within 3 h prior to study drug administration) and at 15, 30, 60, 90 min and 2, 3, 4, 5, 6, 7, 8, 10, 12, and 24 h post study drug administration. Approximately 6 mL of blood was taken into sodium fluoride/potassium oxalate collection tubes, placed on ice, and centrifuged at 4 °C for plasma isolation. The resulting plasma was stored at -70 °C until analysis. The plasma assay was conducted by Frontage Laboratories Inc. (Exton, PA). Salicylic acid and acetylsalicylic acid plasma levels were determined by using a valid High Performance Liquid Chromatography with tandem Mass Spectrometry (LC-MS/MS) method [21]. The validated method has a lower limit of quantitation of 50 ng/mL for salicylic acid and 20 ng/mL for acetylsalicylic acid.

PK parameters were assessed for both acetylsalicylic acid, and its more stable primary metabolite salicylic acid [22]. The primary PK parameters were area under the curve for both the maximal study duration (AUC_0-t_) and extrapolation to infinity (AUC_0-inf_), maximum concentration (C_max_), and time to reach maximum concentration (T_max_). The full list of PK parameters of acetylsalicylic acid and salicylic acid are shown in Supplemental Table 2.

### Pharmacodynamic assessments

Assessments included extent of platelet aggregation following AA and collagen stimuli using LTA and thromboxane B2 (TxB_2_) level measurements [23, 24]. The full list of PD parameters for TxB_2_ are shown in Supplemental Table 3.

Blood samples for platelet aggregation assessments were taken at baseline and at 1, 3, 6, 8, and 24 h post study drug administration and collected in sodium citrate for the measurements of platelet aggregation by LTA in response to conventional cyclooxygenase-1 (COX-1)-related platelet agonists (AA and collagen) after study treatment. Blood samples were centrifuged to prepare platelet rich plasma (PRP) and subsequently, platelet poor plasma (PPP). For the platelet aggregation assay, the PRP sample was incubated for 10 min at 37 °C. Platelet aggregation was induced at 37 °C with constant stirring (1000 RPM) by arachidonic acid (0.5 mg/mL, BioData Corporation, Horsham, PA) or type I fibrillar collagen (4 µg/mL, Chronolog Corporation, Haversham, PA) on an optical platelet aggregometer (Chronolog Corporation, Haversham, PA). The maximal and late (6 min) readings of platelet aggregation were recorded. Each sample was analyzed within 2 h after the sample was taken.

Samples for measurement of serum TxB_2_ concentration were taken at baseline and at 30 min, and 1, 2, 3, 4, 6, 8, 10, and 24 h after study drug administration. For the TxB_2_ assay, samples were incubated immediately (mostly within 1–5 min, and never longer than 30 min from sampling), for 1 h at 37 °C. Serum was obtained and then frozen at − 70 °C until analysis. TxB_2_ assay was conducted by University of Vermont Colchester Research Facility (Colchester, VT) [25]. Serum samples were thawed and a consistent dilution of 1:10 with the ELISA kit (Enzo Life Sciences, Farmington, NY) assay buffer was performed with each sample. Because of a systematic error in sample processing for the TxB_2_ samples that was not discovered until several subjects had been dosed and processed, there were only 17 paired (both study drug doses) and 4 single samples (3 with PL-ASA, 1 with EC-ASA) available for analysis.

All laboratory personnel involved were blinded to treatment allocations.

### Safety assessments

Safety and tolerability were assessed through collection of adverse events (AEs), clinical laboratory assessments, vital signs, and physical examination findings.

### Statistical methods

Four study populations were defined. The Safety Population consisted of all volunteers who were randomized and received any study drug. The next three populations consisted of all subjects who received at least one dose of study drug and did not have any major protocol deviations, and additionally, the PK population consisted of all subjects who had sufficient concentration–time data to calculate at least one key parameter (AUC_0-t_, AUC_0-∞_, or C_max_); the PD LTA population consisted of all subjects who had sufficient platelet aggregation data; and the PD TxB_2_ population consisted of all subjects who had sufficient serum TxB_2_ concentration data.

Summary statistics consisted of frequencies and percentages of responses in each category for discrete measures and of count, means, standard deviations (SDs), coefficient of variations (CVs), medians, minimums and maximums, and geometric mean values for continuous measures, and were presented by treatment.

The PK and PD primary parameters (such as AUCs, T_max_, and C_max_) were determined by implementation of a noncompartmental analysis (NCA) using the validated software program Phoenix WinNonlin Professional version 8.3 (Certara Inc, Mountain View, CA). The PK and PD parameters were calculated by an independent group (Projections Research Inc. Phoenixville, PA).When feasible, AUCs were calculated by a combination of linear trapezoidal methods on concentrations up and logarithmic trapezoidal methods on concentrations going down (Linear Up/Log Down method). In addition, at least two concentration values occurring after T_max_ were needed for calculation of an AUC_0-t_; at least four concentration values, three of which must have occurred after T_max_, were needed for calculation of an AUC_0-∞_. T_max_ and C_max_ were obtained by visual inspection of the concentration–time profiles. A minimum of 3 concentration samples were required to derive T_max_ and C_max_. In the event of two or more identical “peak” concentrations, the earlier value was reported.

The AUCs and C_max_ analyses were performed on log-transformed parameters using a linear mixed-effect, repeated-measures ANOVA model with treatment, period, and sequence as fixed effects and subject as a random effect. The geometric least square mean ratio (GLSMR) of PL-ASA to EC-ASA, 95% CI, and p-value were obtained from model.

As part of PD evaluation, incidence of aspirin response based on various definitions was calculated as number of subjects who met the aspirin response definition divided by a total of subjects who either met or failed the definition. The subjects whose results could not be determined due to missing data were excluded from the denominator. The p-value of the incidence difference between PL-ASA and EC-ASA was determined by using McNemar’s Exact test considering the nature of binary matched-pairs data. The time to achieve an aspirin response was analyzed by using a linear mixed-effect, repeated-measures ANOVA model with treatment, period, and sequence as fixed effects and subject as a random effect. If a subject did not show a response, the time to response was imputed as 24 h.

For PK and PD analyses, unless otherwise specified, a 0.05 significance level were used. Since the study was an exploratory study without any hypothesis testing, no adjustments were made for multiplicity comparisons.

## Results

A total of 36 volunteers were enrolled, dosed with two drugs, and completed the study. Demographic characteristics are listed in Supplemental Table 4. There were 10 males and 26 females, with a mean age of 49 years (median 52 years, range 22–69), reflecting the initial inclusion criteria of > 50 years. A total of 26 were White, 6 were Black and 4 were other designations. The median weight of subjects was 98.25 kg (range 62.6 to 171.0 kg), mean (SD) body mass index (BMI) was 34.49 (8.51) kg/m^2^ (from 21.4 to 62.7 kg/m^2^), with 26 (72.2%) of subjects classified as obese (BMI ≥ 30 kg/m^2^). There were no deaths, serious adverse events or adverse events that led to discontinuation of study drug. All treatment-emergent adverse events were reported recovered/resolved by the end of the study.

### Pharmacokinetic (PK) results

**Fig. 1:**
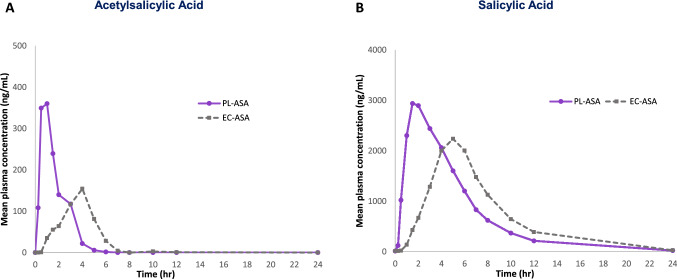
Impact of the pharmaceutical formulation on aspirin’s (81 mg) disposition Plasma concentration time profiles of acetylsalicylic acid (**A**) and salicylic acid (**B**) were compared in fasted volunteers (n = 36) who received single doses of 81 mg PL-ASA and 81 mg EC-ASA.

Values for AUC_0-t_, C_max_ and T_max_ for both acetylsalicylic and salicylic acid demonstrated significantly faster and more complete absorption (i.e., bioavailability) for PL-ASA compared to EC-ASA. As shown in Fig. 1, median time to maximum concentration (T_max_) of acetylsalicylic acid and salicylic acid for PL-ASA were reached approximately 3 h earlier compared to EC-ASA, a difference that was statistically significant (PK parameters are provided in Supplemental Table 5 for acetylsalicylic acid, and Supplemental Tables 6 and 7 for salicylic acid).

The log-normalized ratios of acetylsalicylic acid concentration comparing PL-ASA to EC-ASA are shown in Table 1. Overall, compared to EC-ASA, PL-ASA administration resulted in 44% higher exposure (AUC_0-t_), [Geometric LS mean ratio (GLSMR) = 144%; p = 0.0013], double the maximum concentration (C_max_) (GLSMR = 196%; p < 0.0001).

**Table 1:** Statistical analysis of log-normalized ratio of PL-ASA to EC-ASA for acetylsalicylic acid PK parameters. – PK population (N = 36)

Parameter	PL-ASA	EC-ASA	Ratio (%)	95% CI	p-value
AUC_0-t_ (h*ng/mL)^a^	601 (41.1)	416 (53.7)	144	117–178	0.0013
C_max_ (ng/mL)^a^	720 (33.7)	368 (80.3)	196	148–259	< 0.0001
T_max_ (h)^b^	1.01 (0.47, 3.03)	4.00 (1.50, 6.63)			< 0.0001

The AUC_0-t_ and C_max_ analyses were performed on log-transformed parameters using a linear mixed-effects ANOVA model with treatment, period, and sequence as fixed effects and subject as a random effect

*AUC*_*0-t*_ area under curve from time zero to last measurable concentration, *C*_*max*_ maximum concentration, *CI* confidence interval, *h* hour, *PK* pharmacokinetic

^a^Geometric least square mean (coefficient of variation, %)

^b^Median (range)

For the parameter AUC_0-∞_, PL-ASA also showed higher values for salicylic acid that did not reach statistical significance. Similar comparisons of AUC_0-∞_, for acetylsalicylic acid were inconclusive due to limited sample availability with calculable values, especially during the critical first hour following dosing when acetylsalicylic acid is known to hydrolyze to salicylic acid rapidly within minutes of drug administration (short half-life), and particularly for EC-ASA dosing. As a result, the full characterization of the acetylsalicylic acid PK profiles was impacted, and interpretation of this particular parameter between PL-ASA and EC-ASA was limited.

Overall, the PK results showed that PL-ASA had faster onset and more complete bioavailability than EC-ASA at single dose of 81 mg.

### Pharmacodynamic (PD) results

#### Light transmission aggregometry (LTA)

**Fig. 2:**
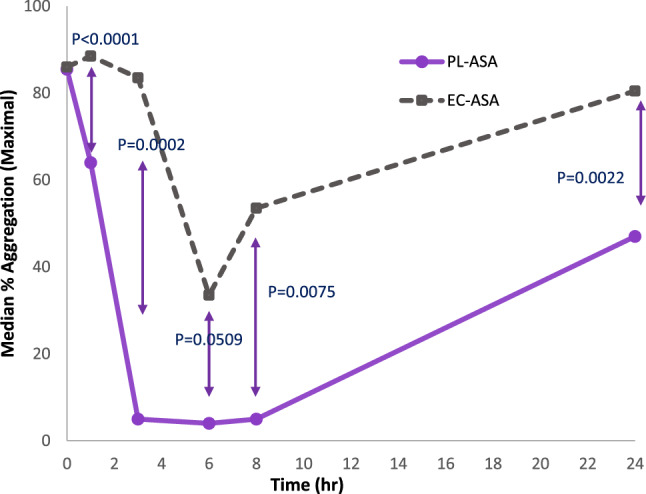
Arachidonic acid-induced platelet aggregation assessed by LTA over time for PL-ASA and EC-ASA**.** Values are medians. p-values were calculated by using mixed-effects, repeated-measure ANOVA model with sequence, period, and treatment as fixed effects and subject as a random effect

Median maximum platelet aggregation values after addition of AA to plasma samples at various timepoints are depicted in Fig. 2. PL-ASA demonstrated significantly faster platelet inhibition and lower residual platelet activity compared to EC-ASA at all measured time points after baseline, with the difference becoming significant within the first hour following study drug administration. At the final assessment time point of 24 h, PL-ASA treatment was associated with approximately 50% residual aggregation, while EC-ASA median residual aggregation was significantly higher at approximately 80% (p = 0.0022). A total of 29 of 36 (80.6%) subjects in the PL-ASA group and 21 of 36 (58.3%) subjects in the EC-ASA group demonstrated < 20% AA-induced platelet aggregation (p = 0.0386). The median time to < 20% residual aggregation was 2.93 h for PL-ASA and 6.18 h for EC-ASA (p = 0.0104).

The observations of higher incidence of responsiveness and faster time to achieve responsiveness in the PL-ASA group were consistent with the approximately 96% higher C_max_ of acetylsalicylic acid and a more complete bioavailability observed for PL-ASA as shown in the PK assessments.

### Thromboxane B2 assessments

Analysis for TxB_2_ was based on 21 subjects, only 17 of which were paired for PL-ASA and EC-ASA dosing, due to a systematic error in sample processing for the TxB_2_ samples that was not discovered until several subjects had been dosed and processed. Among the 4 subjects who only had TxB_2_ data available for one treatment, three received PL-ASA and one received EC-ASA.

**Fig. 3:**
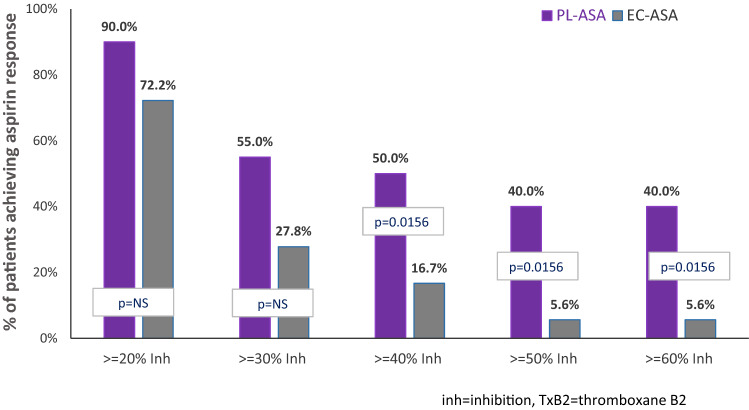
Response to Aspirin: Inhibition of TxB_2_ Production (N = 21) p-values were calculated by using McNemar's Exact test for binary matched-pairs data. The TxB_2_ analysis was based on 21 subjects who had valid TxB_2_ concentration data

**Fig. 4:**
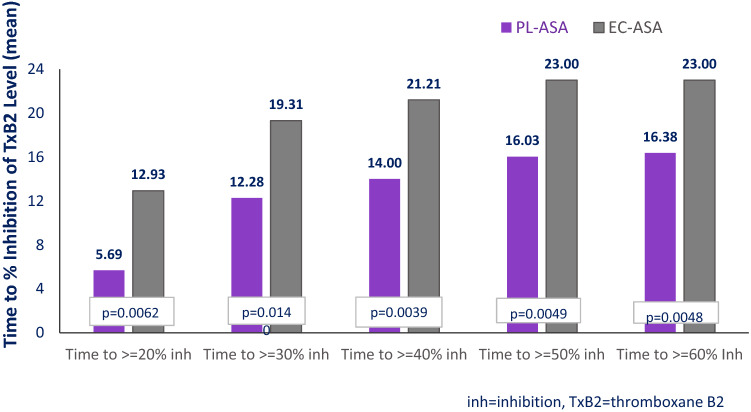
Time to Inhibition of TxB_2_ Production, hours (N = 21). Values are significantly different at all timepoints. p-values were calculated by using mixed-effects, repeated-measure ANOVA model with sequence, period, and treatment as fixed effects and subject as a random effect. The time to cut-off was set as 24 h if the cutoff point was not achieved at 24 h post dose. The TxB_2_ analysis was based on 21 subjects who had valid TxB_2_ concentration data

The median serum TxB_2_ concentration prior to drug administration was 96.53 ng/mL and 100.5 ng/mL for PL-ASA and EC-ASA, respectively, with no baseline difference between the two treatment groups (p = 0.2357). The median serum TxB_2_ concentration dropped to its lowest value of 66.16 ng/mL within 4 h after a single dose of PL-ASA, while the median serum TxB_2_ level only dropped to 85.17 ng/mL after 8 h from the single dose of EC-ASA. Subjects treated with PL-ASA had significantly lower serum TxB_2_ concentration at each post-dose sampling time point (all p-values were < 0.05), compared to those when treated with EC-ASA (Table 2; Fig. 3). Additionally, PL-ASA reached levels of inhibition significantly faster than EC-ASA (Fig. 4).

**Table 2:** Summary of serum TxB_2_ concentration (ng/mL) at each time point—PD TxB_2_ population (N = 21)

Time point median (min, max)	PL-ASA (N = 20)	EC-ASA (N = 18)	p-value
Baseline	96.53 (73.4, 117.3)	100.5 (71.2, 117.2)	0.2357
0.5 h post	96.10 (46.0, 117.4)	101.8 (75.6, 117.3)	0.0287
1 h post	87.84 (26.5, 114.5)	98.27 (68.2, 119.4)	0.0005
2 h post	77.62 (17.8, 97.6)	98.89 (61.8, 114.3)	< 0.0001
3 h post	72.06 (0.8, 93.1)	96.55 (56.7, 116.3)	0.0004
4 h post	66.16 (11.5, 98.6)	95.17 (40.9, 112.9)	0.0001
6 h post	72.92 (17.7, 94.8)	85.25 (11.8, 108.5)	0.0039
8 h post	72.80 (17.8, 99.3)	85.17 (6.3, 110.3)	0.0412
10 h post	65.59 (14.1, 99.7)	89.40 (54.2, 111.0)	0.0007
24 h post	73.66 (37.9, 97.3)	81.49 (39.3, 109.0)	0.0203

p-values were calculated based on a linear nixed-effects ANOVA model with sequence, period and treatment as fixed effects and subject as random effect

*EC* enteric-coated, *PD* pharmacodynamic, *SD* standard deviation, *TxB*_*2*_ thromboxane B_2_

Overall, PL-ASA administration at an 81 mg dose resulted in more rapid, more potent, and more durable platelet inhibition assessed by AA-induced LTA and by TxB_2_ concentrations.

## Discussion

The characteristics of PL-ASA were specifically designed to help protect the GI lining from local injury [17] while reliably delivering therapeutic aspirin levels in a bioequivalent manner to plain, immediate-release aspirin. Enteric-coated formulations were also developed in an attempt to limit gastric injury, however, they have been shown to result in unpredictable and incomplete aspirin absorption [22], underscoring the need to identify novel formulations to overcome such limitations.

The main findings of this prospective, randomized crossover PK-PD study are that a single dose of 81 mg of PL-ASA resulted in rapid and complete absorption. PL-ASA demonstrated faster and more complete absorption resulting in more rapid and durable levels of platelet inhibition compared with EC-ASA. Further, one-third of the subjects, when dosed with EC-ASA did not have quantifiable sample readings within the 24 h period. These findings with 81 mg are consistent with similar published observations with the 325 mg dose [19]. Importantly, because of the crossover design of this study, the differential results observed can be attributed to the differences in formulations rather than to variability of individual responses.

PL-ASA 81 mg provided rapid and predictable platelet inhibition as assessed by both AA-LTA and inhibition of TxB_2_ formation. For AA-LTA, a low level of residual platelet reactivity was observed within hours after administration of PL-ASA, significantly lower than that observed after EC-ASA administration.

Regarding inhibition of TxB_2_ production, aspirin is unique in its irreversible blockade of the COX-1 enzyme, resulting in inhibition for the lifetime of a portion of platelets and therefore, a cumulative effect over several doses as more platelets are inhibited. Although no single dose of 81 mg aspirin produces complete inhibition of TxB_2_, which may take several doses to achieve [24], the greater extent and earlier effect observed with PL-ASA compared to EC-ASA may result in achievement of complete inhibition at an earlier point.

The observed PK-PD advantages of PL-ASA compared to EC-ASA reported in this study are in line with the known erratic and delayed absorption of EC-ASA, as noted in the FDA professional labeling for these formulations [26]. In this study, EC-ASA showed more variability in absorption than PL-ASA, as observed by the higher coefficient of variation (80.3% vs. 33.7%).

The clinical implications of such significant PK-PD differences between formulations are unknown in the absence of randomized trials assessing clinical outcomes, however, in specific treatment settings such differences might be meaningful. For example, faster onset and predictable effect may be beneficial following an interventional stenting procedure, while more durable platelet inhibition may be helpful in patient populations with high rates of pharmacologic aspirin nonresponse, such as patients with diabetes or obesity. Further randomized studies are needed to determine whether clinical outcomes can be improved by the optimized PK-PD profile of this novel aspirin formulation.

## Limitations

This study was conducted in individuals without known ASCVD, consistent with initial determinations of PK profiles, although the median weight of the subjects in this study was high with most of the participants being obese and similar to that of a previous PK-PD study [20] which limits the applicability of the study to a non-obese population. Accordingly, the observations reported here should be interpreted in that context and may not necessarily be applicable to a non-obese population. As this was a single dose study, where the dose would not be expected to achieve steady state, and because of the cumulative effect of daily aspirin on platelet inhibition, caution should be taken in extending these results to longer term dosing. Moreover, our study compared 81 mg PL-ASA vs EC-ASA and not plain aspirin. However, 81 mg plain aspirin is not commonly utilized in real-world practice, and is not currently marketed in the US. PL-ASA 325 mg was previously found to be bioequivalent to plain, immediate-release aspirin in a PK/PD study [19]. The US FDA granted a biowaver (ie, no requirement for a bioequivalence study) for the 81 mg dose because it is dose proportional to the 325 mg dose and because there is no marketed immediate-release 81 mg product with which to conduct a bioequivalence study. Fewer samples for subjects dosed with EC-ASA had calculable PK information with regard to acetylsalicylic acid determinations, likely secondary to the erratic absorption of EC-ASA. While this discrepancy may have affected the ability to compare formulations, it also further underscored the difference between the formulations. Additionally, the error in sample processing for TxB_2_ assessments limits full conclusions from these data. However, full characterization of AA-LTA was available for all samples, which may be helpful and informative to physicians for determination of antiplatelet effect.

## Conclusions

PL-ASA, a novel liquid-filled capsule with a phospholipid-aspirin complex, provides predictable PK and PD effects. PL-ASA results in faster and more complete absorption after a single 81 mg dose compared with EC-ASA. Additionally, PL-ASA provides faster, more potent, and more durable inhibition of platelet aggregation compared with EC-ASA assessed by LTA. The observations of the current study are consistent with previous findings from published comparative studies with the 325 mg dose. Longer term dosing and other studies are needed to fully characterize the PK-PD profile of the 81 mg dose.

## Supplementary Information

Below is the link to the electronic supplementary material.

Supplementary file1 (DOCX 76 kb)
