# Supplementary material for: Pharmacokinetic and pharmacodynamic profiles of a novel phospholipid-aspirin complex liquid formulation and low dose enteric-coated aspirin: results from a prospective, randomized, crossover study
Source: J Thromb Thrombolysis. 2022 Aug 29;54(3):373–81. doi: 10.1007/s11239-022-02687-5 (PMC9421621; doi:10.1007/s11239-022-02687-5)
Supplement: Supplementary file 1 — Supplementary file1 (DOCX 76 kb) [file 11239_2022_2687_MOESM1_ESM.docx]

**Supplement to**

**Pharmacokinetic and Pharmacodynamic Profiles of a Novel Phospholipid-Aspirin Complex Liquid Formulation and Low Dose Enteric Coated Aspirin: Results from a Prospective, Randomized, Crossover Study**

Franchi F, Schneider DJ, Prats J, Fan W, Rollini F, Been L, Taatjes-Sommer HS, Bhatt DL, Deliargyris EN, Angiolillo DJ

**Corresponding Author:**

Dominick J. Angiolillo, MD, PhD,

University of Florida College of Medicine-Jacksonville,

655 West 8th Street, Jacksonville, Florida 32209, United States

Tel: +1-904-244-3933; Fax: +1-904-244-3102

e-mail: [dominick.angiolillo@jax.ufl.edu](mailto:dominick.angiolillo@jax.ufl.edu)

<https://orcid.org/0000-0001-8451-2131>

**Supplemental Figure 1.** Study schematic

**Supplemental Table 1.** Inclusion and Exclusion criteria

**Supplemental Table 2.** Pharmacokinetic Parameters

**Supplemental Table 3.** Pharmacodynamic Parameters

**Supplemental Table 4.** Demographics of the study population

**Supplemental Table 5.** Summary of PK Parameters for Acetylsalicylic Acid in Plasma for PL-ASA vs EC-ASA – PK Population (N=36)

**Supplemental Table 6.** Summary of PK Parameters for Salicylic Acid in Plasma for PL-ASA vs EC-ASA – PK Population (N=36)

**Supplemental Table 7.** Statistical Analysis of Log-normalized Ratio of PL-ASA to EC-ASA for Salicylic Acid PK Parameters. – PK Population (N=36)

**Supplemental Figure 1.** Study Schematic

**Supplemental Table 1.** Inclusion and Exclusion Criteria

| **Inclusion** | **Exclusion** |
| --- | --- |
| - At least 18 years of age and less than 75 years without known medical conditions requiring treatment; - If female, a negative pregnancy test and not nursing; - If female and of childbearing potential, using adequate birth control for the duration of the study - Non-smoker, for at least 3 months prior to screening; - Consumes on average no more than 2 alcoholic drinks per day for at least 30 days prior to screening; - Agrees to refrain from alcohol for 48 hours prior to and 48 hours after drug administration - Informed consent | - Significant abnormal screening/baseline laboratory parameters; - Positive urine alcohol/ drug screen result; - Use of any prescription medications other than hormone replacement therapy, thyroid replacement therapy, or oral contraceptives <3 days prior to study drug administration; - Use of antacid medications, including over-the-counter (OTC) products <24 hours prior to study drug administration; - Use of dietary or herbal supplements containing salicylates, fish oil, or any vitamins <2 weeks of study drug administration; - Use of any of the following medications <2 weeks prior to study drug administration:   - Nonsteroidal anti-inflammatory drugs (NSAIDs), including aspirin;   - Any antiplatelet agent;   - Any anticoagulant agent; - Use of an investigational agent <30 days prior to drug administration; - Hypersensitivity or contraindications to aspirin, ibuprofen, or other NSAID; - Soy allergy or sensitivity; - History of:   - Gastrointestinal problems including ulcers, frequent indigestion, or frequent heartburn;   - Coronary arterial disease, stroke, or congestive heart failure;   - Asthma, nasal polyps, or angioedema other than resolved childhood asthma;   - Kidney or liver disease;   - Thrombocytopenia, neutropenia, bleeding disorder, or history of non-trauma related hemorrhage;   - Chronic hypertension; - Subject’s platelets aggregation unresponsive to arachidonic acid (defined as <60% of aggregation); - History of cancer < 5 years |

**Supplemental Table 2.** Pharmacokinetic Parameters

| **Parameter** | **Definition** |
| --- | --- |
| AUC_0-t (SA)_ | Area under the concentration-time curve of plasma salicylic acid from time zero until the last quantifiable value |
| AUC_0-∞ (SA)_ | Area under the concentration-time curve of plasma salicylic acid from time zero until the infinity |
| C_max (SA)_ | Observed maximum concentration of plasma salicylic acid |
| T_max (SA)_ | Sampling time of observed maximum concentration of plasma salicylic acid |
| t_1/2 (SA)_ | Terminal elimination half-life of plasma salicylic acid |
| λ_z (SA)_ | Terminal elimination rate constant of plasma salicylic acid |
| V_D_/F _(SA)_ | Apparent volume of distribution of plasma salicylic acid |
| CL/F _(SA)_ | Apparent total clearance of the drug from plasma salicylic acid |
| AUC_0-t (ASA)_ | Area under the concentration-time curve of plasma acetylsalicylic acid from time zero until the last quantifiable value |
| AUC_0-∞ (ASA)_ | Area under the concentration-time curve of plasma acetylsalicylic acid from time zero until the infinity |
| C_max (ASA)_ | Observed maximum concentration of plasma acetylsalicylic acid |
| T_max (ASA)_ | Sampling time of observed maximum concentration of plasma acetylsalicylic acid |
| t_1/2 (ASA)_ | Terminal elimination half-life of plasma acetylsalicylic acid |
| λ_z (ASA)_ | Terminal elimination rate constant of plasma acetylsalicylic acid |
| V_D_/F _(ASA)_ | Apparent volume of distribution of plasma acetylsalicylic acid |
| CL/F _(ASA)_ | Apparent total clearance of the drug from plasma acetylsalicylic acid |

**Supplemental Table 3.** Pharmacodynamic Parameters

| **Parameter** | **Definition** |
| --- | --- |
| I _(ITxB2)_ | % of inhibition of serum TxB_2_ from baseline at each time point |
| I_max_ _(ITxB2)_ | Observed maximum percent inhibition of serum TxB2 |
| T_max_ _(ITxB2)_ | \| Time to maximum percent inhibition of serum TxB2 \| \| --- \| |
| AUC_0-24_ _(ITxB2)_ | Area under percent inhibition of serum TxB2 taken directly from the inhibition time course profile through the 24-hour sample |
|  |  |
| C_min (TxB2)_ | Observed minimum serum TxB_2_ concentration up to 24 hours after study drug |
| T_min (TxB2)_ | Time to minimum serum TxB_2_ concentration up to 24 hours after study drug |
| AUC_0-24_ _(TxB2)_ | Area under the concentration-time curve of serum TxB2 from the time zero until the 24-hour sample |
|  |  |
| C_min (AA-LTA)_ | Observed minimum AA-induced platelet aggregation |
| T_min (AA-LTA)_ | Time to minimum AA-induced platelet aggregation |

**Supplemental Table 4.** Demographics of the study population

| **Parameter** |  | **N=36** |
| --- | --- | --- |
| Age | Mean, SD  Median, (min, max) | 49.0 (12.0)  52.0 (22, 69) |
| Sex, n (%) | Female  Male | 26 (72.2%)  10 (27.8%) |
| Race, n (%) | Asian  Black  Hispanic  Other  White/Caucasian | 1 (2.8%)  6 (16.7%)  2 (5.6%)  1 (2.8%)  26 (72.2%) |
| Weight (kg) | Mean, SD  Median, (min, max) | 98.15 (21.74)  98.25 (62.6, 171.0) |
| BMI (kg/m^2^) | Mean, SD  Median, (min, max) | 34.49 (8.51)  32.96 (21.4, 62.7) |

**Supplemental Table 5.** Summary of PK Parameters for Acetylsalicylic Acid in Plasma for PL-ASA vs EC-ASA – PK Population (N=36)

|  | **PL-ASA** | | | |  | **EC-ASA** | | | |
| --- | --- | --- | --- | --- | --- | --- | --- | --- | --- |
| **Parameter (unit)** | **N** | **Mean** | **SD** | **Median** |  | **N** | **Mean** | **SD** | **Median** |
| C_max_ (ng/mL) | 34 | 756 | 238 | 763 |  | 23 | 447 | 259 | 362 |
| T_max_ (h) | 34 | 1.18 | 0.753 | 1.01 |  | 23 | 3.52 | 1.37 | 4.00 |
| AUC_0-t_ (h*ng/mL) | 34 | 645 | 267 | 565 |  | 23 | 474 | 199 | 453 |
| AUC_0-inf_ (h*ng/mL) | 8 | 770 | 256 | 685 |  | 0 | N/A | N/A | N/A |
| Λ_z_ (1/h) | 8 | 2.18 | 0.474 | 2.16 |  | 0 | N/A | N/A | N/A |
| T_1/2_ (h) | 8 | 0.331 | 0.0692 | 0.322 |  | 0 | N/A | N/A | N/A |
| CL/F (L/h) | 8 | 2.50 | 1.66 | 2.08 |  | 0 | N/A | N/A | N/A |
| V_d_/F (L) | 8 | 114 | 33.3 | 118 |  | 0 | N/A | N/A | N/A |

AUC_0-inf_=area under the plasma concentration-time curve from time zero to infinity; AUC_0-t_=area under the plasma concentration-time curve from time zero until the last quantifiable value; C_max_=observed maximum plasma concentration; CL/F=apparent clearance; EC=enteric-coated; N/A= not available; PK=pharmacokinetic; SD=standard deviation; T_1/2_=terminal elimination half-life; T_max_=time to maximum plasma concentration; V_D_/F=apparent volume of distribution; λ_z_=terminal elimination constant.

**Supplemental Table 6.** Summary of PK Parameters for Salicylic Acid in Plasma for PL-ASA vs EC-ASA – PK Population (N=36)

|  | **PL-ASA** | | | |  | **EC-ASA** | | | |
| --- | --- | --- | --- | --- | --- | --- | --- | --- | --- |
| **Parameter (unit)** | **N** | **Mean** | **SD** | **Median** |  | **N** | **Mean** | **SD** | **Median** |
| C_max_ (ng/mL) | 36 | 4200 | 932 | 4150 |  | 36 | 3160 | 1210 | 3070 |
| T_max_ (h) | 36 | 1.92 | 1.25 | 1.52 |  | 36 | 4.75 | 2.01 | 4.89 |
| AUC_0-t_ (h*ng/mL) | 36 | 15800 | 5810 | 14500 |  | 36 | 14000 | 4940 | 13100 |
| AUC_0-inf_ (h*ng/mL) | 36 | 16200 | 5950 | 14900 |  | 36 | 15000 | 5160 | 13700 |
| Λ_z_ (1/h) | 36 | 0.300 | 0.118 | 0.319 |  | 33 | 0.302 | 0.107 | 0.332 |
| T_1/2_ (h) | 36 | 2.93 | 1.84 | 2.17 |  | 33 | 2.74 | 1.41 | 2.09 |
| CL/F (L/h) | 36 | 2.73 | 1.42 | 2.44 |  | 33 | 5.39 | 3.80 | 3.78 |
| V_D_/F (L) | 36 | 5.64 | 2.03 | 5.44 |  | 33 | 6.03 | 2.15 | 5.91 |

AUC_0-inf_=area under curve from time zero extrapolated to infinite time; AUC_0-t_=area under curve from time zero to last measurable concentration; C_max_=maximum concentration; CL/F=apparent clearance ; EC=enteric coated; h=hour; max=maximum; min=minimum; PK=pharmacokinetic; SD=standard deviation; T_1/2_=terminal elimination phase half-life; T_max_=time to maximum concentration; V_D_/F= apparent volume of distribution; λ_Z_=terminal elimination rate consistent.

**Supplemental Table 7.** Statistical Analysis of Log-normalized Ratio of PL-ASA to EC-ASA for Salicylic Acid PK Parameters. – PK Population (N=36)

| **Parameter** | **PL-ASA** | **EC-ASA** | **Ratio (%)** | | **95% CI** | **p-value** |
| --- | --- | --- | --- | --- | --- | --- |
| AUC_0-t_ (h*ng/mL)^a^ | 14800 (36.7) | 13100 (38.1) | | 113 | 102 – 125 | 0.0210 |
| AUC_0-∞_ (h*ng/mL)^a^ | 15300 (37.0) | 14300 (35.2) | | 107 | 96 – 118 | 0.21 |
| C_max_ (ng/mL)^a^ | 4100 (23.7) | 2940 (41.0) | | 139 | 121 – 160 | <0.0001 |
| T_max_(h)^b^ | 1.52 (0.55, 5.92) | 4.89 (2.0, 11.9) | |  |  | <0.0001 |

AUC_0-inf_=area under curve from time zero extrapolated to infinite time;  AUC_0-t_=area under curve from time zero to last measurable concentration; C_max_=maximum concentration; CI=confidence interval; CV=coefficient of variation; h=hour; LS=least square; PK=pharmacokinetic.

^a^ Geometric LS mean (CV%); ^b^median (range)
Notes: The AUCs and C_max_ analyses were performed on log-transformed parameters using a linear mixed-effects ANOVA model with treatment, period, and sequence as fixed effects and subject as a random effect.
